# Evaluation of Reference Genes for Normalization of Gene Expression Using Quantitative RT-PCR under Aluminum, Cadmium, and Heat Stresses in Soybean

**DOI:** 10.1371/journal.pone.0168965

**Published:** 2017-01-03

**Authors:** Mengmeng Gao, Yaping Liu, Xiao Ma, Qin Shuai, Junyi Gai, Yan Li

**Affiliations:** National Key Laboratory of Crop Genetics and Germplasm Enhancement, National Center for Soybean Improvement, Key Laboratory for Biology and Genetic Improvement of Soybean (General, Ministry of Agriculture), Jiangsu Collaborative Innovation Center for Modern Crop Production, Nanjing Agricultural University, Nanjing, Jiangsu, China; Chinese University of Hong Kong, HONG KONG

## Abstract

Quantitative reverse transcription polymerase chain reaction (qRT-PCR) is widely used to analyze the relative gene expression level, however, the accuracy of qRT-PCR is greatly affected by the stability of reference genes, which is tissue- and environment- dependent. Therefore, choosing the most stable reference gene in a specific tissue and environment is critical to interpret gene expression patterns. Aluminum (Al), cadmium (Cd), and heat stresses are three important abiotic factors limiting soybean (*Glycine max*) production in southern China. To identify the suitable reference genes for normalizing the expression levels of target genes by qRT-PCR in soybean response to Al, Cd and heat stresses, we studied the expression stability of ten commonly used housekeeping genes in soybean roots and leaves under these three abiotic stresses, using five approaches, BestKeeper, Delta Ct, geNorm, NormFinder and RefFinder. We found *TUA4* is the most stable reference gene in soybean root tips under Al stress. Under Cd stress, *Fbox* and *UKN2* are the most stable reference genes in roots and leaves, respectively, while *60S* is the most suitable reference gene when analyzing both roots and leaves together. For heat stress, *TUA4* and *UKN2* are the most stable housekeeping genes in roots and leaves, respectively, and *UKN2* is the best reference gene for analysis of roots and leaves together. To validate the reference genes, we quantified the relative expression levels of six target genes that were involved in soybean response to Al, Cd or heat stresses, respectively. The expression patterns of these target genes differed between using the most and least stable reference genes, suggesting the selection of a suitable reference gene is critical for gene expression studies.

## Introduction

Quantitative reverse transcription polymerase chain reaction (qRT-PCR) is one of the most widely used techniques to detect the changes in gene expression [1], due to its relatively accurate quantification, high sensitivity and high throughput. The difference in the amount and quality of the template can affect the efficiency of the qRT-PCR reactions [2], therefore it is essential to normalize the expression level of the target gene by using a reference gene as an internal control. In general, an ideal reference gene should demonstrate a consistent expression level across all tested tissues or conditions [3].

Housekeeping genes are commonly used as the reference genes for qRT-PCR, such as 18S ribosomal RNA (*18S* rRNA), 25S ribosomal RNA (*25S* rRNA), β-actin (*ACT11*), cyclophilin (*CYP2*), elongation factor 1-alpha (*ELF1A*), glyceraldehyde-3-phosphate dehydrogenase (*GAPDH*), translation elongation factor (*TEF*), tubulin (*TUB4*) and polyubiquitin (*UBQ*) [4]. The housekeeping genes are involved in basic metabolic processes and important for normal cell growth, therefore their expression levels are thought to be stable [5–7]. However, many recent studies found that the expression levels of housekeeping genes may vary largely across different tissues, developmental stages, or experimental conditions [6–9]. Therefore, it is necessary to select stably expressed reference genes before they are utilized for normalizing the expression levels of target genes by qRT-PCR.

Soybean is an economically important crop worldwide, which provides important source of vegetable oil and proteins [10]. The production and quality of soybean can be affected by a variety of adverse environmental conditions, including abiotic and biotic stresses. Analyzing gene expression under stresses is important to select the candidate genes for soybean tolerance to various stresses. To date, several studies have been conducted to select stable reference genes in different tissues and under various environments in soybean, including different photoperiods [6], abscisic acid (ABA) treatment [8], as well as drought and salinity stresses [8,9], and the reference genes showed tissue- and stress-dependent [6,8,9].

Aluminum (Al) toxicity, cadmium (Cd) pollution, and heat stresses are three major adverse environments affecting agricultural production in southern China. Al toxicity is a major factor limiting crop production on acidic soils [11, 12]. The primary symptom of Al toxicity is the inhibition of root growth which later disrupts water and nutrient uptake by the roots [13]. Cd is a widespread toxic heavy metal pollutant in agricultural soils [14]. Cd can accumulate in human kidneys, leading to loss of calcium and osteoporosis [15]. Several studies found that Cd concentrations in soybean grains exceeded the maximum permissible levels in Japan [16], Argentina [17], and China [18], which poses potential threats to food safety. The damage of Cd toxicity to soybean includes inhibition of root and shoot growth, less water and nutrient uptake, chlorosis, and decreased yield [18–21]. It has been found that warming increased Cd uptake and translocation in rice seedlings [22], which suggests Cd would be an important environmental stress in warmer regions and under future warmer climates. A recent study found that heat waves during early pod development in soybean caused significant yield loss [23]. Therefore, Al, Cd, and heat stresses are three major factors limiting soybean production especially under warmer climates. However, there are limited studies on gene expression analysis and selection of reference genes for qRT-PCR in soybean under Al, Cd, and heat stresses. In this study, we did a comprehensive analysis of ten commonly used reference genes in soybean to select the most stable ones for normalization of gene expression by qRT-PCR under Al, Cd, and heat stresses, which would be helpful to improve the accuracy of gene expression analysis under these important abiotic stresses.

## Materials and Methods

### Plant materials, growth conditions and stress treatments

The seeds of soybean (*Glycine max* [L.] Merr.) cultivar Kefeng-1 used in this study are provided by National Center for Soybean Improvement (Nanjing, China). The experiments were conducted in plant growth chambers with a 14 h/10 h (light /dark) cycle at 26°C / 24°C (light /dark) and 50–70% relative humidity. Soybean seeds were germinated in sterile sand for three days in dark. For Al toxicity treatment, 3-day-old seedlings were transferred to 0.5 mM CaCl_2_ (pH = 4.3) for one day, then to 0.5 mM CaCl_2_ solution (pH = 4.3) containing 25 μM AlCl_3_ (Al stress) or without Al (control). The primary root tips (0–1 cm) of ten seedlings were collected at 6, 12 and 24 h for Al stress and control, respectively [24]. For Cd and heat treatments, the soybean seedlings were transferred to containers filled with 1/2 Hoagland solution (pH = 5.8), which was replaced every three days. For Cd treatment, 14-day-old seedlings were treated with 100 μM CdCl_2_ (Cd stress) in 1/2 Hoagland solution or 1/2 Hoagland solution without Cd (control), and samples were collected after 3, 12 and 24 h, according to the previous studies [25–26]. For heat stress, 14-day-old seedlings were maintained at 42°C for 1, 3 and 6 h as described previously [27]. The seedlings grown under normal conditions at the corresponding time points were used as control. For each biological replicate, the root tips (0–1 cm) from 10 individual plants were collected and pooled for each root sample, and the newest fully expanded trifoliolate leaves from three individual plants were harvested and pooled together for each leaf sample. Leaf and root samples were collected separately, frozen quickly in liquid nitrogen and stored at -80°C for RNA extraction. All experiments were conducted with three biological replications. There are 30 samples for each replication and 90 samples in total for this study. For Al stress, we collected 18 root samples (3 time points x 2 treatments x 3 replicates = 18). For Cd stress, we collected 18 root samples and 18 leaf samples, and 18 root samples and 18 leaf samples for heat stress as well.

### RNA extraction and cDNA synthesis

Total RNA was isolated using RNAprep Pure Plant Kit (TianGen, China) according to the manufacturer’s instruction. Electrophoresis with 1% agarose gel was used to determine the integrity of total RNA. The quality and concentrations of RNA were measured by Infinite M200 (Tecan, Switzerland). Frist strand cDNA was synthesized by reverse transcription of 1 μg total RNA with PrimeScript^™^ RT reagent Kit with gDNA Eraser (Perfect Real Time) (TaKaRa, Japan), in a volume of 20 μl according to manufacturer’s protocol. All cDNA samples were stored at -20°C for later use.

### qRT-PCR assays

Primers from published literature with good specificity and amplification efficiency were utilized in our study (S1 Table). All primers were synthesized by Invitrogen (Shanghai, China). Quantitative RT-PCR experiments were carried out using SYBR^®^ Premix Ex Taq^™^ (Tli RNaseH Plus) (TaKaRa, Japan) on LightCycler 480 (Roche, Switzerland). The program of the qRT-PCR was 95°C for 5 min, followed by 40 cycles at 95°C for 10 s, 60°C for 15 s and 72°C for 15 s. Dissociation curves were obtained using a thermal melting profile performed after the last PCR cycle: 95°C decreases to 40°C at the speed of 5°C per second followed by a constant increase in the temperature between 60°C and 95°C. The MIQE (Minimum Information for Publication of Quantitative Real-Time PCR Experiments) guidelines proposed the use of quantification cycle (Cq) value over the threshold cycle (Ct) according to the RDML (Real-Time PCR Data Markup Language) data standard [2]. Cq value represents the number of cycles when the density of fluorescence meets the set threshold. Therefore, Cq values were used in this study. Each sample was tested in three technical replicates.

A series of 10-fold dilutions of cDNA templates (10–1,000 fold dilution) were made to generate standard curves, and the gene specific amplification efficiency for each primer pair in qRT-PCR was determined by the slope of the log-linear portion of the calibration curve. The gene specific PCR amplification efficiency (E) is calculated by using the equation: E (%) = (10^−1/slope^-1) × 100% [9]. The relative expression levels of target genes were calculated using the 2^-ΔΔCT^ method [28].

### Analysis of the stabilities of reference genes

The stabilities of reference genes were analyzed using software tools, including geNorm [29], BestKeeper [30], NormFinder [31], Delta Ct [32], and RefFinder [33], following the corresponding instructions. The geNorm program identifies the most stable reference genes based on the average pairwise variation of a reference gene with other housekeeping genes, and ranks the reference genes by their expression stability values (M). In general, the lower the M value, the higher is the expression stability of candidate genes [29]. BestKeeper determines the “optimal” reference genes on the basis of pair-wise correlation analysis of all pairs of candidate reference genes [30]. NormFinder calculates the overall variation of the candidate reference genes in all samples and also the variation of intra- and inter-groups [31]. Delta Ct compares the relative expression of pairs of candidate genes within each sample. If the ΔCt value between two reference genes does not change among different samples, it indicates either both genes have stable expression patterns or they are co-regulated among the samples, yet the different ΔCt value suggests that at least one of them is variably expressed. Then the third, fourth, or more genes are introduced into the comparisons to find out which pairs show less variability, and hence which gene has stable expression among the samples tested. Ultimately, an appropriate reference gene can be selected for a particular experimental system [32]. Finally, RefFinder generates a comprehensive ranking by calculating the geometric mean of each reference gene in the above four methods, in which the smaller the ranking, the more stable is the reference gene [33–34].

### Statistical analysis

Statistical analyses were performed using SPSS version 17.0 software. Differences in the relative expression levels of target genes between using the most and two least stable reference genes were analyzed based on Duncan’s multiple range test.

## Results

### Primer specificity and qRT-PCR amplification efficiency

In this study, we chose ten candidate reference genes in soybean whose stabilities have been previously tested under various conditions [6, 8, 9] but not under Al, Cd, or heat stresses. These ten candidate reference genes include *60s*, *ABC*, *ACT11*, *ACT2/7*, *CYP2*, *ELF1A*, *Fbox*, *TUA4*, *TUB4*, and *UKN2* [6, 8, 9]. In order to verify the performance of the candidate reference genes identified in this study, six target genes were selected for qRT-PCR to study their gene expression under Al, Cd and heat stresses, in which *GmALMT1* [35] and *GmARI1* [36] have been reported to play roles in soybean tolerance to Al toxicity, *GmHMA13* and *GmHMA19* were related to Cd stress [37], and *GmGBP1* and *GmHsfA1* were responsive to heat stress [27, 38]. The details of the ten reference genes and six target genes are listed in Table 1.

**Table 1:** The ten candidate reference genes and six target genes used in this study.

Gene	NCBI Accession No.	Gene ID (W82.a2.v1)	Annotation
*60S*	LOC100778077	Glyma.13g318800	60S ribosome protein
*ABC*	LOC100783869	Glyma.12g020500	ATP-binding cassette transporter
*ACT11*	LOC100792119	Glyma.18g290800	Actin 11
*ACT2/7*	LOC100807341	Glyma.19g147900	Actin 7
*CYP2*	LOC106795232	Glyma.12g024700	Cyclophilin
*ELF1A*	LOC100785429	Glyma.19g052400	Elongation factor 1-alpha
*Fbox*	LOC100809876	Glyma.12g051100	Fbox protein
*TUA4*	LOC100781185	Glyma.20g136000	Tubulin alpha
*TUB4*	LOC100798849	Glyma.19g127700	Tubulin beta
*UKN2*	LOC100789577	Glyma.06g038500	Hypothetical protein
*GmALMT1*	LOC100170704	Glyma.03g202200	Aluminum-activated malate transporter
*GmARI1*	LOC100784195	Glyma.11g129000	Ariadne-like E3 ubiquitin ligase
*GmHMA13*	LOC100815324	Glyma.09g055600	Heavy metal ATPase
*GmHMA19*	LOC100776309	Glyma.17g166800	Heavy metal ATPase
*GmGBP1*	LOC732608	Glyma.01g008600	GAMYB-binding protein
*GmHsfA1*	LOC732544	Glyma.16g091800	Heat shock transcription factor

The specificity of the amplification was shown by the melting curves in qRT-PCR (S1 Fig). A single peak of each melting curve in the qRT-PCR experiments indicated good specificity of the primers. The qRT-PCR amplification efficiency (E) and correlation coefficient (R^2^) were calculated based on the slope of the calibration curves (Table 2). For the ten candidate reference genes and six target genes, the E values were 93.74% to 104.80% and R^2^ ranged from 0.9977 to 0.9999 (Table 2). The results showed that all 16 pairs of primers met the requirement of qRT-PCR experiments.

**Table 2:** Amplification characteristics of the ten candidate reference genes and six target genes in qRT-PCR.

Gene	Amplicon length (bp)	Efficiency (%)	Correlation coefficient (R^2^)
*60S*	125	100.70	0.9999
*ABC*	106	97.16	0.9994
*ACT11*	142	96.91	0.9992
*ACT2/7*	119	96.72	0.9984
*CYP2*	130	93.74	0.9995
*ELF1A*	162	96.44	0.9997
*Fbox*	93	101.90	0.9990
*TUA4*	159	93.91	0.9991
*TUB4*	137	99.40	0.9999
*UKN2*	156	101.45	0.9999
*GmALMT1*	195	104.80	0.9982
*GmARI1*	150	102.93	0.9989
*GmHMA13*	182	97.34	0.9992
*GmHMA19*	166	98.76	0.9989
*GmGBP1*	194	100.24	0.9977
*GmHsfA1*	491	99.68	0.9994

### Expression profiling of ten candidate reference genes

**Fig 1:**
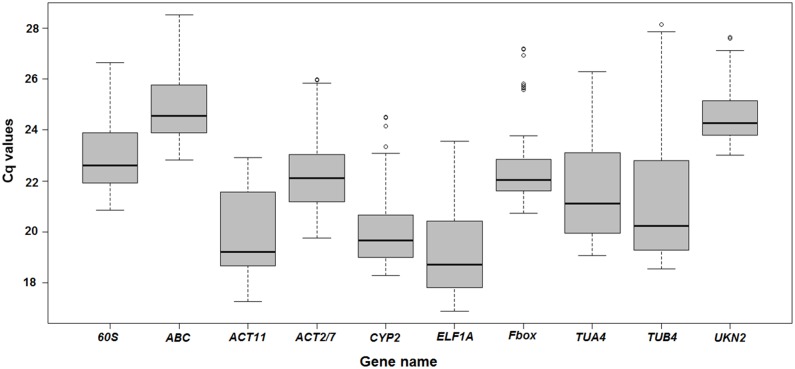
Distribution of quantification cycle (Cq) values for ten candidate reference genes in all samples. Expression levels of the ten candidate reference genes were examined in all the samples in this study and shown by the boxplots. The solid line within each box represents the 50^th^ percentile. The lower boundary and upper boundary of each box represents the 25^th^ and 75^th^ percentile, respectively. The line above and below the vertical dashed lines represents the maximum and minimum values, respectively. The circles represents potential outliers.

The transcript abundance of the ten candidate reference genes in all samples (including Al, Cd, and heat treatments and controls) was determined by the Cq values from qRT-PCR experiments (Fig 1). Cq is the number of cycles at which the fluorescence exceeds the detection threshold. During the PCR amplification, a cDNA sample with more abundance reaches the threshold at a lower Cq value, corresponding to a higher gene expression level. The Cq values of the ten candidate reference genes ranged from 16.88 to 28.53, with an average Cq value of 21.93. All of the Cq values were within the valid range of qRT-PCR, demonstrating their feasibility for further analysis. The mean Cq value of each candidate reference gene varied from 19.19 to 24.85 (Fig 1). *ELF1A* was the most abundant reference gene in this study, indicated by its mean Cq value of 19.19 (minimum mean Cq value in the set), whereas *ABC* was the least abundant reference gene (whose mean Cq value was 24.85).

### Expression stability of the candidate reference genes in all samples

**Fig 2:**
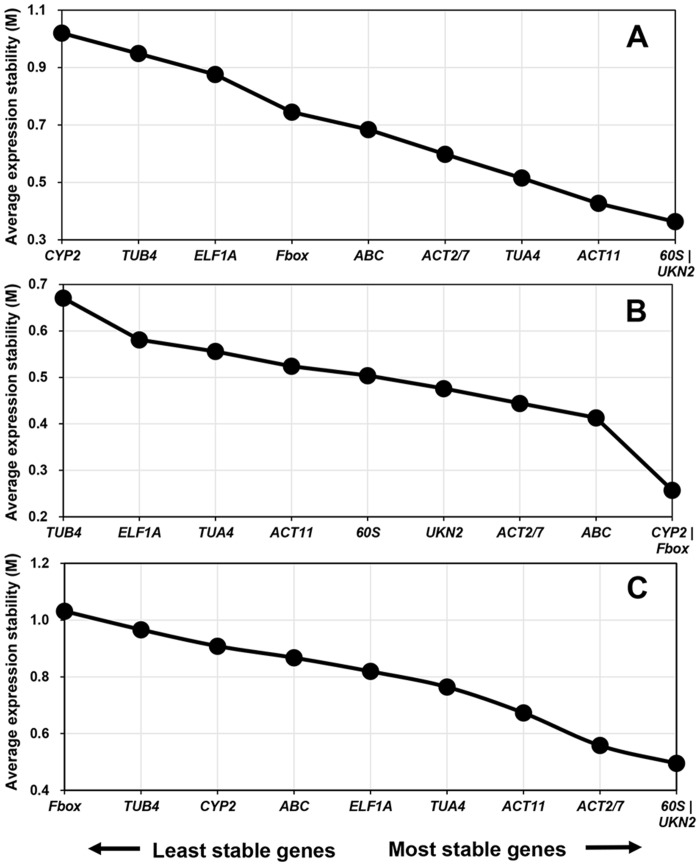
The expression stability of the ten soybean reference genes across all treatments in this study. Y-axis represents the average expression stability (M) values analyzed by geNorm. (A) Leaf samples, (B) Root samples, (C) Leaf and root samples together.

To identify the suitable reference genes for qRT-PCR that could be used in gene expression analysis under Al, Cd, and heat stresses, the expression profiles of the ten candidate reference genes in soybean roots and/or leaves across all experiments in this study were analyzed together using RefFinder, which gives a comprehensive evaluation of the reference genes based on the rankings made by the four algorithms, BestKeeper, Delta Ct, geNorm, and NormFinder. For leaf samples, the top five most stable reference genes identified by RefFinder were *UKN2* > *ACT11* > *60S* > *TUA4* > *ACT2/7* (Table 3), which also showed low M values (the lowest five) by geNorm analysis (Fig 2A), implying their expression stability in leaves, and *CYP2* was ranked as the most unstable gene by both RefFinder (Table 3) and geNorm (Fig 2A). For root samples, the top five most stably expressed genes were *Fbox* > *ACT2/7* > *CYP2* > *ABC* > *UKN2* as shown by RefFinder (Table 3), which were also the five most stable genes identified by geNorm (Fig 2B). *TUB4* was found as the least stable gene in roots by RefFinder (Table 3) and geNorm (Fig 2B). When samples of roots and leaves were analyzed together, *UKN2* and *60S* were identified as the most stably expressed reference genes by RefFinder (Table 3) and geNorm analysis (Fig 2C), whereas *TUB4* was ranked as the least stable reference gene by RefFinder (Table 3).

**Table 3:** Rankings of the expression stability of ten candidate reference genes by RefFinder.

Ranking	All treatments			Al	Cd			42°C		
	Leaves	Roots	L&R	Roots	Leaves	Roots	L&R	Leaves	Roots	L&R
1	*UKN2*	*Fbox*	*UKN2*	*TUA4*	*UKN2*	*Fbox*	*60S*	*UKN2*	*TUA4*	*UKN2*
2	*ACT11*	*ACT2/7*	*60S*	*ACT11*	*ACT11*	*ACT2/7*	*UKN2*	*60S*	*ACT2/7*	*ACT2/7*
3	*60S*	*CYP2*	*ACT2/7*	*Fbox*	*60S*	*60S*	*ACT2/7*	*TUA4*	*ELF1A*	*60S*
4	*TUA4*	*ABC*	*ACT11*	*UKN2*	*TUA4*	*CYP2*	*ACT11*	*ACT11*	*UKN2*	*ABC*
5	*ACT2/7*	*UKN2*	*ABC*	*TUB4*	*ACT2/7*	*ABC*	*TUA4*	*ACT2/7*	*ABC*	*ELF1A*
6	*ABC*	*ACT11*	*ELF1A*	*60S*	*TUB4*	*UKN2*	*CYP2*	*ABC*	*TUB4*	*ACT11*
7	*ELF1A*	*60S*	*Fbox*	*ELF1A*	*ABC*	*ACT11*	*ABC*	*ELF1A*	*CYP2*	*Fbox*
8	*TUB4*	*TUA4*	*TUA4*	*ACT2/7*	*CYP2*	*TUA4*	*ELF1A*	*Fbox*	*ACT11*	*TUA4*
9	*Fbox*	*ELF1A*	*CYP2*	*ABC*	*ELF1A*	*ELF1A*	*Fbox*	*TUB4*	*60S*	*CYP2*
10	*CYP2*	*TUB4*	*TUB4*	*CYP2*	*Fbox*	*TUB4*	*TUB4*	*CYP2*	*Fbox*	*TUB4*

### Expression stability of the candidate reference genes under Al toxicity

Root is the primary tissue influenced by Al toxicity, which inhibits crop growth and yield in acid soils. Therefore we try to identify the most stable reference genes in soybean roots under Al toxicity. *TUA4* was listed on the top of the ranking by RefFinder (Table 3), also by geNorm, BestKeeper and Delta Ct (S2 Fig, S2 Table). However, NormFinder found that *ACT11* had a better performance than the other 9 candidate reference genes, followed by *TUA4* and *TUB4* (S2 Table). *CYP2* was consistently evaluated as the least stable reference genes by RefFinder (Table 3), as well as Delta Ct, geNorm and NormFinder (S2 Fig, S2 Table). Therefore, when studying the relative expression of genes involved in soybean response to Al toxicity, *TUA4* should be used as the reference gene while *CYP2* should be avoided.

### Expression stability of the candidate reference genes under Cd stress

Studies have demonstrated that Cd pollution in soil can have an adverse effect on soybean growth [39]. It is important to find stable reference genes for normalizing gene expression of target genes under Cd stress. In the leaves, the most stable reference genes were *UKN2*, *ACT11* and *60S* by RefFinder analysis (Table 3), and *Fbox* was the least stable one in the ranking (S3 Fig, S3 Table). But in the roots, *Fbox* was the most stable gene by RefFinder followed by *ACT2/7* and *60S* (Table 3), and *TUB4* was consistently ranked as the least stable gene by all approaches (S3 Fig, S4 Table). When both leaves and roots were analyzed together, *60S*, *UKN2* and *ACT2/7* were identified as the top three stable reference genes under Cd stress by RefFinder (Table 3) and the other four methods (S3 Fig, S5 Table). Meanwhile, *Fbox* and *TUB4* were found to be the least stable ones by RefFinder (Table 3) and three methods including geNorm (S3 Fig, S5 Table). These results revealed that a reference gene might perform highly variable among different tissues.

### Expression stability of the candidate reference genes under heat stress

Next we searched for the most suitable reference genes to be used for gene expression analysis under 42°C heat stress. *UKN2* and *60S* were the top two stably expressed genes in leaves under heat stress (Table 3 and S6 Table). If considering the roots under heat stress, *TUA4* was the best one to be chosen as demonstrated by RefFinder, NormFinder, and Delta Ct (Table 3 and S7 Table), and *ACT2/7* and *ELF1A* were the other two reference genes with good stability in roots (Table 3 and S7 Table). The four evaluation methods consistently showed that *UKN2* was the most stable reference gene in both leaves and roots (Table 3 and S8 Table). The top two most stable genes in both roots and leaves were *UKN2* and *ACT2/7* ranked by both RefFinder (Table 3) and geNorm (S4 Fig).

### Validation of the reference genes identified in this study

**Fig 3:**
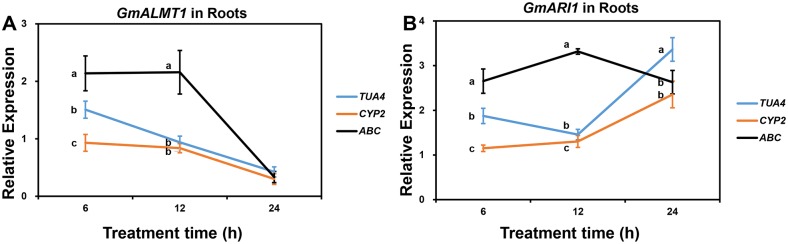
Relative expression levels of *GmALMT1* (A) and *GmARI1* (B) in soybean roots under 25 μM AlCl_3_ (pH4.3) treatment normalized by the most (*TUA4*) and two least (*CYP2* and *ABC*) stable reference genes. The relative expression levels of two target genes relative to the control samples at corresponding time points were calculated using the 2^-ΔΔCT^ method. Bars represent the mean ± SE (standard error) from three replications. Different letters represent significant difference (*P* < 0.05) at each time point based on Duncan’s multiple range test.

**Fig 4:**
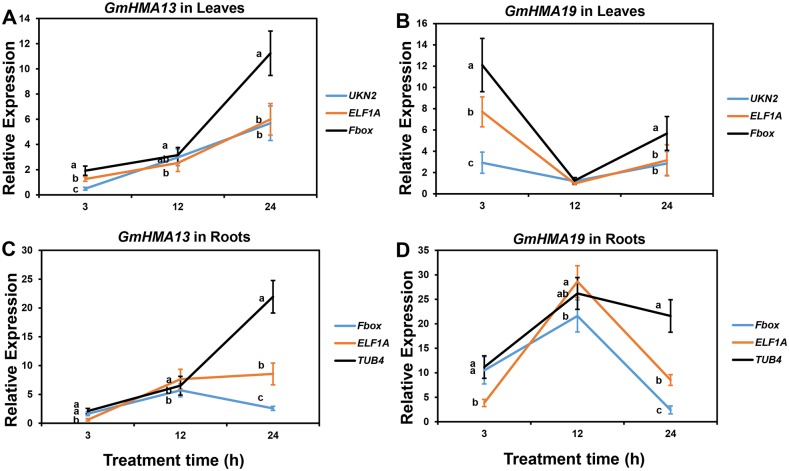
Relative expression levels of *GmHMA13* and *GmHMA19* in soybean leaves and roots under 100 μM CdCl_2_ treatment normalized by the most (*UKN2*) and two least (*ELF1A* and *Fbox*) stable reference genes in leaves and the most (*Fbox*) and two least (*ELF1A* and *TUB4*) stable reference genes in roots. (A) *GmHMA13* in soybean leaves, (B) *GmHMA19* in soybean leaves, (C) *GmHMA13* in soybean roots, and (D) *GmHMA19* in soybean roots. The relative expression levels of two target genes relative to the control samples at corresponding time points were calculated using the 2^-ΔΔCT^ method. Bars represent the mean ± SE (standard error) from three replications. Different letters represent significant difference (*P* < 0.05) at each time point based on Duncan’s multiple range test.

**Fig 5:**
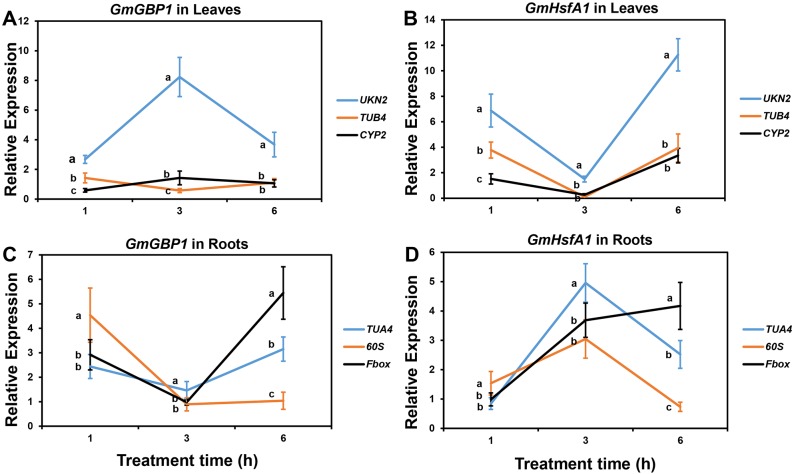
Relative expression levels of *GmGBP1* and *GmHsfA1* in soybean leaves and roots under 42°C heat stress normalized by the most (*UKN2*) and two least (*TUB4* and *CYP2*) stable reference genes in leaves and the most (*TUA4*) and two least (*60S* and *Fbox*) stable reference genes in roots. (A) *GmGBP1* in soybean leaves, (B) *GmHsfA1* in soybean leaves, (C) *GmGBP1* in soybean roots, and (D) *GmHsfA1* in soybean roots. The relative expression levels of two target genes relative to the control samples at corresponding time points were calculated using the 2^-ΔΔCT^ method. Bars represent the mean ± SE (standard error) from three replications. Different letters represent significant difference (*P* < 0.05) at each time point based on Duncan’s multiple range test.

To validate the reference genes identified in this study, we analyzed the expression of six target genes in soybean, in which *GmALMT1* and *GmARI1* were considered to be involved in soybean response to Al toxicity [35–36], *GmHMA13* and *GmHMA19* participated in soybean response to Cd stress [37], and *GmGBP1* and *GmHsfA1* were responsive to heat stress in soybean [27, 38]. As determined by RefFinder, we chose the most stable reference gene, *TUA4*, and two least stable genes, *CYP2* and *ABC*, to normalize the expression levels of the two target genes under Al toxicity. As shown in Fig 3A, the relative expression level of *GmALMT1* was up-regulated by 25 μM AlCl_3_ at 6 and 12 h when normalized by *TUA4* gene, which was significantly different (*P* < 0.05 based on Duncan’s multiple range tests) with the relative expression levels when using *CYP2* or *ABC* as the reference gene. Similar situation occurred in *GmARI1* expression profile (Fig 3B). Compared with *TUA4* as the reference gene, the relative expression levels of *GmARI1* was significantly different (*P* < 0.05) with that when normalized by *CYP2* or *ABC*, and the peak time point of *GmARI1* expression level also shifted when using *ABC* as the reference gene (Fig 3B). Under Cd stress, the most stable reference gene is *UKN2* and the two least stable reference genes are *ELF1A* and *Fbox* in soybean leaves, while *Fbox*, *ELF1A* and *TUB4* were the most and two least stable reference genes in soybean roots. As shown in Fig 4, in both leaves and roots, the two target genes exhibited different expression profiles when using different reference genes. The relative expression levels of both target genes in soybean under Cd stress tend to be inflated when normalized by the least stable reference gene *Fbox* in leaves (Fig 4A and 4B) or *TUB4* in roots (Fig 4C and 4D). Under heat stress, in soybean leaves, the relative expression levels of *GmGBP1* was up-regulated when normalized by the most stable reference gene *UKN2*, which is significantly (*P* < 0.05) different compared with using the two least stable reference genes *TUB4* and *CYP2* at all time-points (Fig 5A). Similar result was found for the target gene *GmHsfA1* (Fig 5B). In soybean roots, the most stable reference gene *TUA4* and the two least stable reference genes *60S* and *Fbox* were selected to normalize the expression levels of target genes under heat stress. The relative expression patterns of *GmGBP1* and *GmHsfA1* were different when normalized by *TUA4* compared with the two least stable reference genes (Fig 5C and 5D). These results highlight that selection of a suitable reference gene is critical for gene expression studies.

## Discussion

Climate changes and human activities have generated many adverse environments for plants, such as acid rain, accumulation of heavy metals in soil, drought, high temperature, and salinity. Seeking for the molecular mechanisms and candidate genes are important to improve the abilities of plants to resist or tolerate these stresses. Analysis of gene expression patterns under abiotic stresses helps understanding the functions and regulation of genes. qRT-PCR is an efficient and most recognized method to evaluate the transcript abundance of genes. Many studies have demonstrated that the selection of suitable reference genes is critical to interpret the qRT-PCR results, and there is no reference gene showing stable expression in different tissues across all environments [6, 8, 9, 40–42]. In soybean, the stable reference genes for study of gene expression under drought and salinity stresses have been reported [8, 9], but the suitable reference genes for study of gene expression under Al toxicity, Cd and heat stresses are needed to be explored.

In this study, ten candidate reference genes, *60S*, *ABC*, *ACT11*, *ACT2/7*, *CYP2*, *ELF1A*, *Fbox*, *TUA4*, *TUB4* and *UKN2*, were selected for normalization of gene expression under Al toxicity, Cd and heat stresses. The ten primer pairs for all candidate reference genes showed good amplification efficiencies of 93.74 ~ 104.80% (Table 2), and the average Cq value of each gene ranged from 19.19 to 24.85, indicating the qRT-PCR results are suitable for further analysis. Five approaches, geNorm, BestKeeper, NormFinder, delta Ct, and RefFinder, were employed to analyze the expression stability of these reference genes. Although the rank order of the reference genes was not identical when using different approaches, the overall most and least stable reference genes are consistent among several statistical analysis. The web-based tool RefFinder integrates the four statistical algorithms including geNorm, BestKeeper, NormFinder, and delta Ct, to rank the overall stability of candidate reference genes. Therefore, we selected the suitable reference genes mainly based on the output from RefFinder. When the samples from all three stresses (Al, Cd and heat stresses) were analyzed together, *UKN2* was identified as the overall most stably expressed reference gene by RefFinder (Table 3). Under Al toxicity, *TUA4* performed as the most stable reference gene in soybean roots, followed by *ACT11*, *Fbox* and *UKN2*. For Cd stress, *60S* and *UKN2* are the optimal reference genes when analyze the gene expression in both leaves and roots. And *UKN2* also showed highest stability in both leaves and roots under 42°C heat stress. There is no report on selection of reference genes for Al or heat stress in soybean to date. A previous study showed that *ACT3*, *PP2A*, *ELF1B* and *F-box* were the most stable reference genes in soybean under Cd stress based on geNorm and NormFinder analysis [43], while *60S* and *UKN2*, the most stable reference genes in soybean under Cd stress found in this study, were not evaluated in the previous report [43].

We also used six previously reported genes that are responsive to Al, Cd and heat stresses, respectively, to validate their relative expression using the most and least stable reference genes identified in our study. The results showed that the expression patterns of target genes differed between using the most and least stable reference genes (Figs 3–5), suggesting that selection of a suitable reference gene is critical for gene expression studies. This study provides a list of suitable reference genes for normalization of gene expression in soybean under Al toxicity, Cd and heat stresses, which would help to accurately analyze the gene expression patterns under these important abiotic stresses.

## Supporting Information

S1 Fig Melting curves of ten candidate reference genes and six target genes. (A) to (J) represent ten candidate reference genes, *60S*, *ABC*, *ACT11*, *ACT2/7*, *CYP2*, *ELF1A*, *Fbox*, *TUA4*, *TUB4* and *UKN2*, respectively. (K) to (P) represent the six target genes, *GmALMT1*, *GmARI1*, *GmHMA13*, *GmHMA19*, *GmGBP1* and *GmHsfA1*, respectively.
(TIF)

S2 Fig The expression stability of the ten reference genes in soybean roots under 25 μM AlCl_3_ (pH4.3) treatment. Y-axis represents the average expression stability (M) values analyzed by geNorm.
(TIF)

S3 Fig The expression stability of the ten reference genes in soybean under 100 μM CdCl_2_ treatment. Y-axis represents the average expression stability (M) values analyzed by geNorm. (A) Leaf samples, (B) Root samples, (C) Leaf and root samples together.
(TIF)

S4 Fig The expression stability of the ten reference genes in soybean leaves and roots under 42°C heat stress. Y-axis represents the average expression stability (M) values analyzed by geNorm.
(TIF)

S1 Table Primers of ten candidate reference genes and six target genes for qRT-PCR in this study. (DOCX)

S2 Table Rankings and expression stability values of ten candidate reference genes in soybean roots under 25 μM AlCl_3_ (pH4.3) treatment. From top to the bottom represent the most stable to least stable gene.
(DOCX)

S3 Table Rankings and expression stability values of ten candidate reference genes in soybean leaves under 100 μM CdCl_2_ treatment. From top to the bottom represent the most stable to least stable gene.
(DOCX)

S4 Table Rankings and expression stability values of ten candidate reference genes in soybean roots under 100 μM CdCl_2_ treatment. From top to the bottom represent the most stable to least stable gene.
(DOCX)

S5 Table Rankings and expression stability values of ten candidate reference genes in soybean leaves and roots under 100 μM CdCl_2_ treatment. From top to the bottom represent the most stable to least stable gene.
(DOCX)

S6 Table Rankings and expression stability values of ten candidate reference genes in soybean leaves under 42°C heat stress. From top to the bottom represent the most stable to least stable gene.
(DOCX)

S7 Table Rankings and expression stability values of ten candidate reference genes in soybean roots under 42°C heat stress. From top to the bottom represent the most stable to least stable gene.
(DOCX)

S8 Table Rankings and expression stability values of ten candidate reference genes in soybean leaves and roots under 42°C heat stress. From top to the bottom represent the most stable to least stable gene.
(DOCX)
