# Supplementary figures and images for: Evaluation of Reference Genes for Normalization of Gene Expression Using Quantitative RT-PCR under Aluminum, Cadmium, and Heat Stresses in Soybean
Source: PLoS One. 2017 Jan 3;12(1):e0168965. doi: 10.1371/journal.pone.0168965 (PMC5207429; doi:10.1371/journal.pone.0168965)

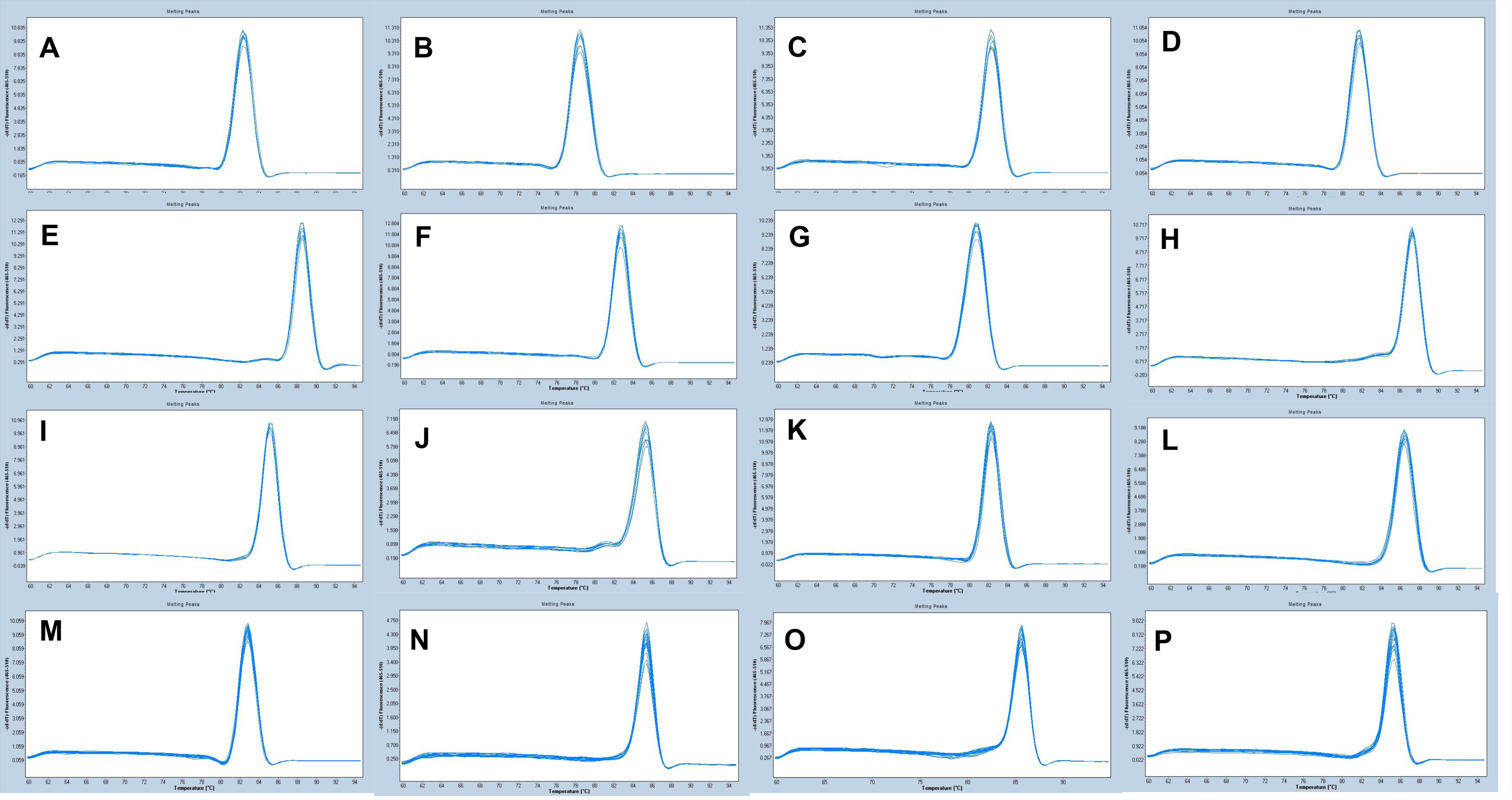

Supplement: S1 Fig — (A) to (J) represent ten candidate reference genes, 60S, ABC, ACT11, ACT2/7, CYP2, ELF1A, Fbox, TUA4, TUB4 and UKN2, respectively. (K) to (P) represent the six target genes, GmALMT1, GmARI1, GmHMA13, GmHMA19, GmGBP1 and GmHsfA1, respectively. (TIF) [file pone.0168965.s001.tif]

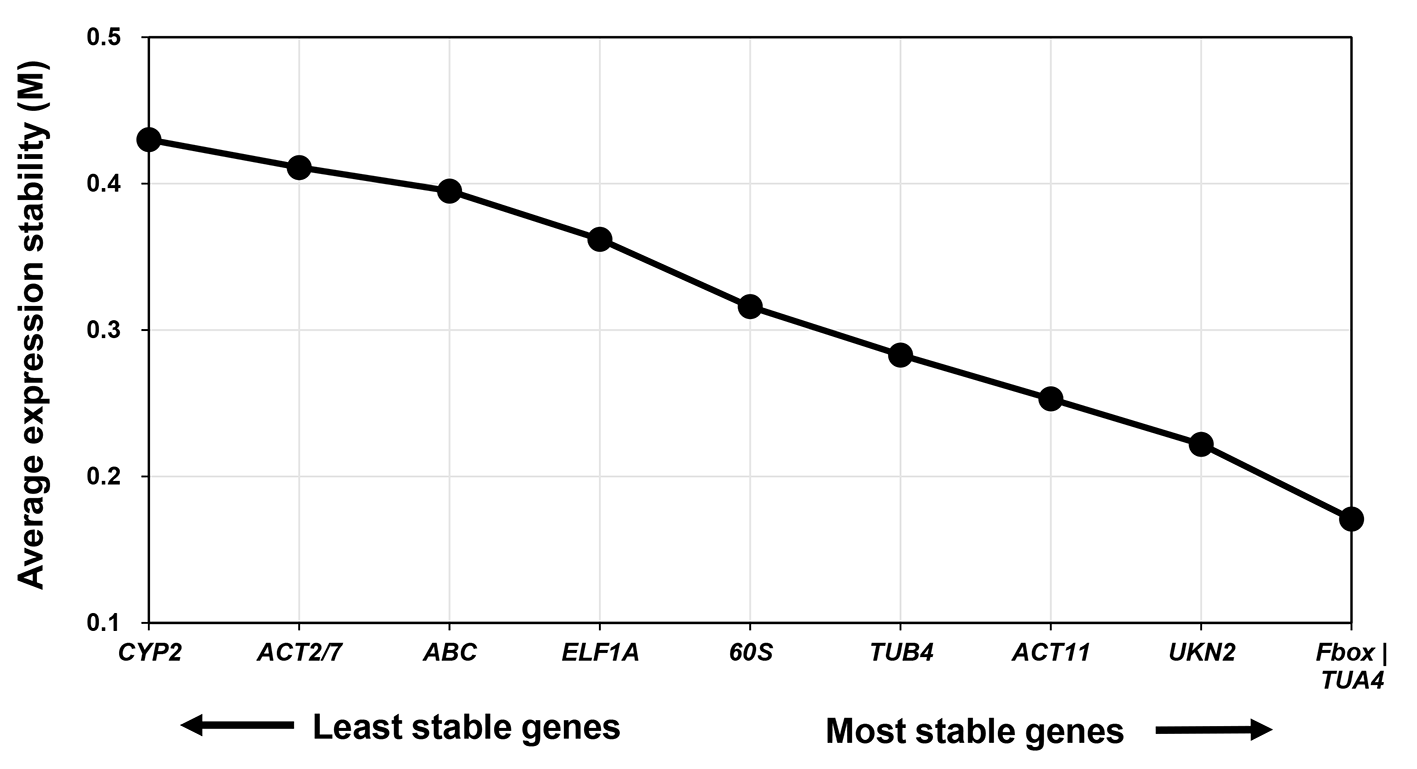

Supplement: S2 Fig — Y-axis represents the average expression stability (M) values analyzed by geNorm. (TIF) [file pone.0168965.s002.tif]

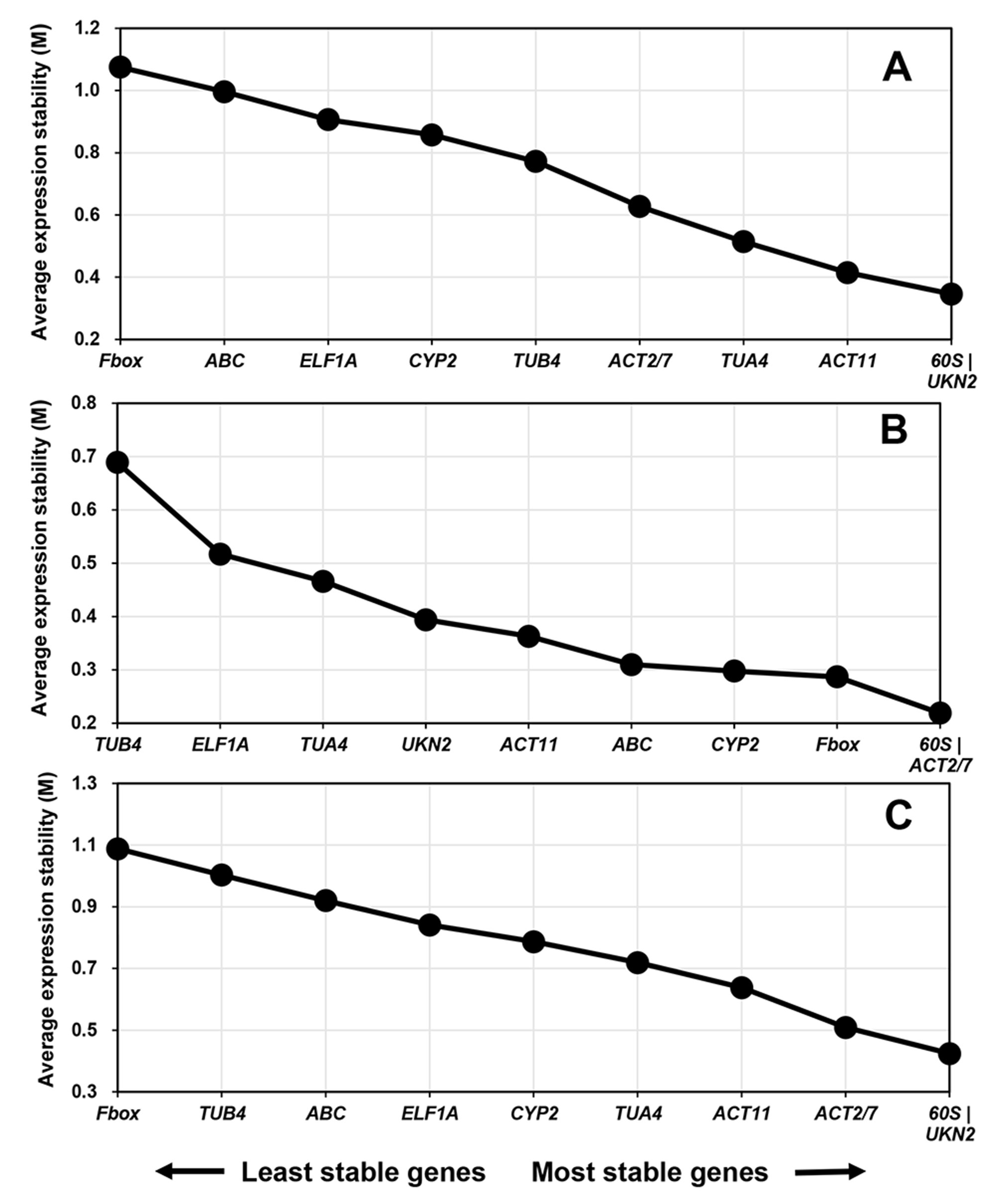

Supplement: S3 Fig — Y-axis represents the average expression stability (M) values analyzed by geNorm. (A) Leaf samples, (B) Root samples, (C) Leaf and root samples together. (TIF) [file pone.0168965.s003.tif]

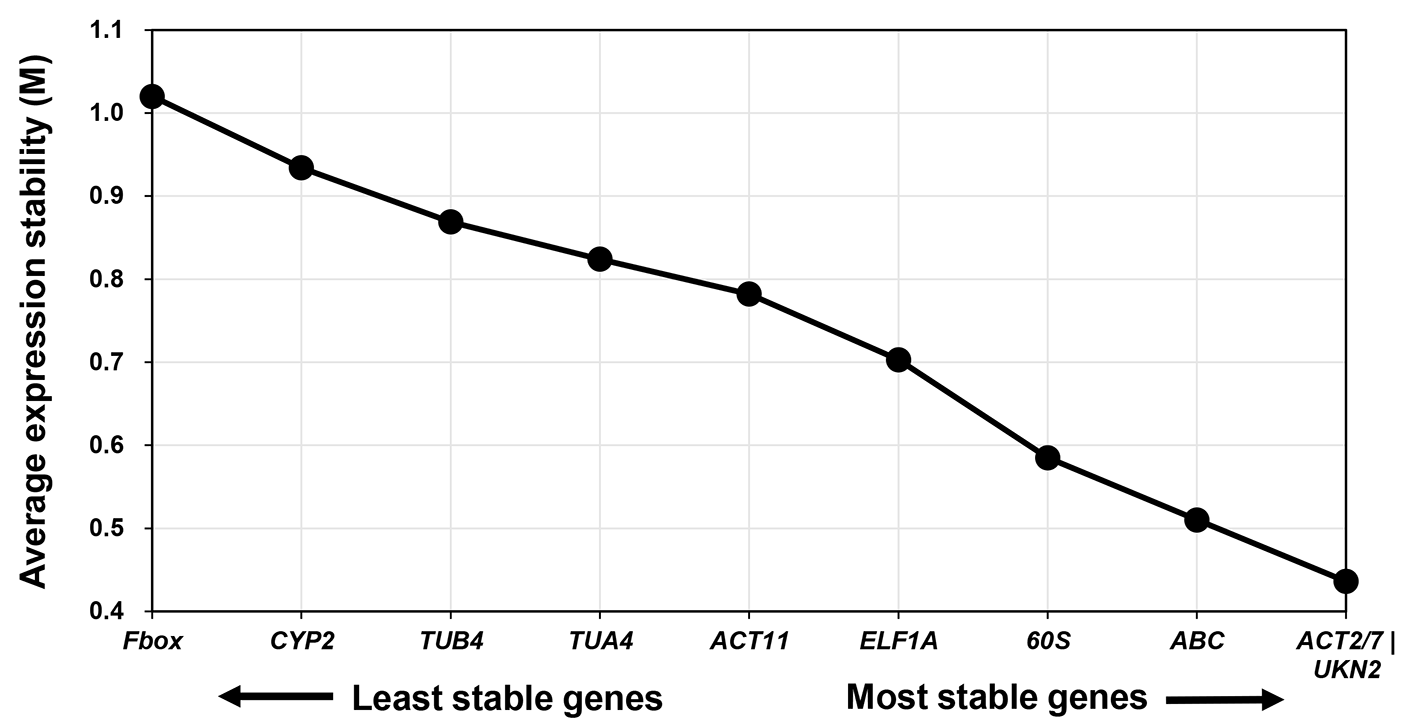

Supplement: S4 Fig — Y-axis represents the average expression stability (M) values analyzed by geNorm. (TIF) [file pone.0168965.s004.tif]
