# Supplementary material for: Evaluation of Reference Genes for Normalization of Gene Expression Using Quantitative RT-PCR under Aluminum, Cadmium, and Heat Stresses in Soybean
Source: PLoS One. 2017 Jan 3;12(1):e0168965. doi: 10.1371/journal.pone.0168965 (PMC5207429; doi:10.1371/journal.pone.0168965)
Supplement: S1 Table — (DOCX) [file pone.0168965.s005.docx]

**S1 Table.** **Primers of ten candidate reference genes and six target genes for qRT-PCR in this study.**

| **Gene** | **Forward primers (5'–3')** | **Reverse primers (5'–3')** | **Reference** |
| --- | --- | --- | --- |
| *60s* | AAAGTGGACCAAGGCATATCGTCG | TCAGGACATTCTCCGCAAGATTCC | 8 |
| *ABC* | CCGCCACCATTCAGATTATGT | GATCAGCAATTATGCACAACG | 8 |
| *ACT11* | CGGTGGTTCTATCTTGGCATC | GTCTTTCGCTTCAATAACCCTA | 6, 8 |
| *ACT2/7* | CTTCCCTCAGCACCTTCCAA | GGTCCAGCTTTCACACTCCAT | 6, 8, 9 |
| *CYP2* | CGGGACCAGTGTGCTTCTTCA | CCCCTCCACTACAAAGGCTCG | 9 |
| *ELF1A* | GACCTTCTTCGTTTCTCGCA | CGAACCTCTCAATCACACGC | 6, 8, 9 |
| *Fbox* | AGATAGGGAAATTGTGCAGGT | CTAATGGCAATTGCAGCTCTC | 8 |
| *TUA4* | CATACCCTAGAATCCATTTC | TGTACTTTCCGTGACGAG | 9 |
| *TUB4* | TGGCGTCCACATTCATTG | GAACTCCATCTCGTCCAT | 9 |
| *UKN2* | TGTGCTCTGTGAAGAGATTG | TCATAATCTGTGTGCAGTTC | 9 |
| *GmALMT1* | GAGCACTTACTCGGGAATGTG | GGACTTTGGCAGTTGATGGG | 35 |
| *GmARI1* | CGCTGGTTCCTGAATTTCCCTTG | GTCGTCGTCGCTGTAGTAGTCC | 36 |
| *GmHMA13* | GAGTCACAAATAGCCGATGC | ACAGAACCAAGTGCCAACC | 37 |
| *GmHMA19* | CTGCGTCGCTGAATCCT | TGCCAGGTCCATCCATCT | 37 |
| *GmGBP1* | TTTGTGAAGGAGAGTAGGGAGGAG | TTAGTAGAGGCCATACCAAGAGCA | 38 |
| *GmHsfA1* | CTTCTCCAGCTTCGTTCGCC | TACCCTCCTGCTTAAGCCGG | 27 |
