# Supplementary material for: Evaluation of Reference Genes for Normalization of Gene Expression Using Quantitative RT-PCR under Aluminum, Cadmium, and Heat Stresses in Soybean
Source: PLoS One. 2017 Jan 3;12(1):e0168965. doi: 10.1371/journal.pone.0168965 (PMC5207429; doi:10.1371/journal.pone.0168965)
Supplement: S2 Table — From top to the bottom represent the most stable to least stable gene. (DOCX) [file pone.0168965.s006.docx]

**S2 Table. Rankings and expression stability values of ten candidate reference genes in soybean roots under 25 μM AlCl_3_ (pH4.3) treatment.** From top to the bottom represent the most stable to least stable gene.

| **RefFinder** | | **BestKeeper** | | **NormFinder** | | **Delta Ct** | | **geNorm (M)** | |
| --- | --- | --- | --- | --- | --- | --- | --- | --- | --- |
| *TUA4* | 1.190 | *TUA4* | 0.194 | *ACT11* | 0.104 | *TUA4* | 0.340 | *TUA4* | 0.171 |
| *ACT11* | 2.510 | *Fbox* | 0.253 | *TUA4* | 0.140 | *ACT11* | 0.350 | *Fbox* | 0.171 |
| *Fbox* | 2.660 | *UKN2* | 0.270 | *TUB4* | 0.209 | *TUB4* | 0.380 | *UKN2* | 0.222 |
| *UKN2* | 3.460 | *TUB4* | 0.309 | *UKN2* | 0.247 | *UKN2* | 0.390 | *ACT11* | 0.253 |
| *TUB4* | 3.660 | *ACT11* | 0.334 | *Fbox* | 0.278 | *Fbox* | 0.400 | *TUB4* | 0.283 |
| *60S* | 6.480 | *60S* | 0.373 | *ELF1A* | 0.358 | *ELF1A* | 0.460 | *60S* | 0.316 |
| *ELF1A* | 6.700 | *CYP2* | 0.403 | *60S* | 0.379 | *60S* | 0.480 | *ELF1A* | 0.362 |
| *ACT2/7* | 8.710 | *ELF1A* | 0.427 | *ACT2/7* | 0.413 | *ACT2/7* | 0.500 | *ABC* | 0.395 |
| *ABC* | 8.740 | *ABC* | 0.477 | *ABC* | 0.420 | *ABC* | 0.500 | *ACT2/7* | 0.411 |
| *CYP2* | 9.150 | *ACT2/7* | 0.489 | *CYP2* | 0.436 | *CYP2* | 0.510 | *CYP2* | 0.430 |
