# Supplementary material for: Evaluation of Reference Genes for Normalization of Gene Expression Using Quantitative RT-PCR under Aluminum, Cadmium, and Heat Stresses in Soybean
Source: PLoS One. 2017 Jan 3;12(1):e0168965. doi: 10.1371/journal.pone.0168965 (PMC5207429; doi:10.1371/journal.pone.0168965)
Supplement: S3 Table — From top to the bottom represent the most stable to least stable gene. (DOCX) [file pone.0168965.s007.docx]

**S3 Table.** **Rankings and expression stability values of ten candidate reference genes in soybean leaves under 100 μM CdCl_2_ treatment.** From top to the bottom represent the most stable to least stable gene.

| RefFinder | | BestKeeper | | NormFinder | | Delta Ct | | geNorm(M) | |
| --- | --- | --- | --- | --- | --- | --- | --- | --- | --- |
| *UKN2* | 1.000 | *UKN2* | 0.231 | *UKN2* | 0.173 | *UKN2* | 0.790 | *60S* | 0.346 |
| *ACT11* | 2.210 | *ACT11* | 0.360 | *ACT11* | 0.192 | *ACT11* | 0.810 | *UKN2* | 0.346 |
| *60S* | 2.280 | *60S* | 0.379 | *60S* | 0.302 | *60S* | 0.860 | *ACT11* | 0.415 |
| *TUA4* | 4.230 | *ACT2/7* | 0.439 | *TUA4* | 0.567 | *TUA4* | 0.980 | *TUA4* | 0.514 |
| *ACT2/7* | 4.730 | *TUA4* | 0.484 | *ACT2/7* | 0.672 | *ACT2/7* | 1.040 | *ACT2/7* | 0.627 |
| *TUB4* | 6.240 | *ABC* | 0.777 | *TUB4* | 0.918 | *TUB4* | 1.150 | *TUB4* | 0.772 |
| *ABC* | 7.420 | *TUB4* | 0.828 | *ABC* | 1.047 | *CYP2* | 1.220 | *CYP2* | 0.857 |
| *CYP2* | 7.710 | *ELF1A* | 0.991 | *CYP2* | 1.054 | *ABC* | 1.230 | *ELF1A* | 0.906 |
| *ELF1A* | 8.490 | *CYP2* | 1.010 | *ELF1A* | 1.127 | *ELF1A* | 1.280 | *ABC* | 0.996 |
| *Fbox* | 10.00 | *Fbox* | 1.032 | *Fbox* | 1.281 | *Fbox* | 1.390 | *Fbox* | 1.075 |
