# Supplementary material for: Evaluation of Reference Genes for Normalization of Gene Expression Using Quantitative RT-PCR under Aluminum, Cadmium, and Heat Stresses in Soybean
Source: PLoS One. 2017 Jan 3;12(1):e0168965. doi: 10.1371/journal.pone.0168965 (PMC5207429; doi:10.1371/journal.pone.0168965)
Supplement: S4 Table — From top to the bottom represent the most stable to least stable gene. (DOCX) [file pone.0168965.s008.docx]

**S4 Table.** **Rankings and expression stability values of ten candidate reference genes in soybean roots under 100 μM CdCl_2_ treatment.** From top to the bottom represent the most stable to least stable gene.

| **RefFinder** | | **BestKeeper** | | **NormFinder** | | **Delta Ct** | | **geNorm(M)** | |
| --- | --- | --- | --- | --- | --- | --- | --- | --- | --- |
| *Fbox* | 2.060 | *UKN2* | 1.367 | *Fbox* | 0.100 | *Fbox* | 0.510 | *60S* | 0.219 |
| *ACT2/7* | 2.590 | *ACT11* | 1.452 | *CYP2* | 0.101 | *CYP2* | 0.530 | *ACT2/7* | 0.219 |
| *60S* | 2.630 | *60S* | 1.567 | *ACT2/7* | 0.228 | *ACT2/7* | 0.530 | *Fbox* | 0.287 |
| *CYP2* | 3.250 | *ABC* | 1.617 | *60S* | 0.300 | *60S* | 0.550 | *CYP2* | 0.298 |
| *ABC* | 4.730 | *ACT2/7* | 1.633 | *ABC* | 0.344 | *ABC* | 0.570 | *ABC* | 0.310 |
| *UKN2* | 4.740 | *Fbox* | 1.788 | *TUA4* | 0.397 | *TUA4* | 0.660 | *ACT11* | 0.363 |
| *ACT11* | 5.090 | *CYP2* | 1.804 | *ELF1A* | 0.534 | *ACT11* | 0.690 | *UKN2* | 0.394 |
| *TUA4* | 6.930 | *TUA4* | 2.167 | *ACT11* | 0.547 | *UKN2* | 0.730 | *TUA4* | 0.466 |
| *ELF1A* | 8.450 | *ELF1A* | 2.216 | *UKN2* | 0.639 | *ELF1A* | 0.740 | *ELF1A* | 0.517 |
| *TUB4* | 10.00 | *TUB4* | 2.693 | *TUB4* | 1.344 | *TUB4* | 1.380 | *TUB4* | 0.689 |
