# Supplementary material for: Evaluation of Reference Genes for Normalization of Gene Expression Using Quantitative RT-PCR under Aluminum, Cadmium, and Heat Stresses in Soybean
Source: PLoS One. 2017 Jan 3;12(1):e0168965. doi: 10.1371/journal.pone.0168965 (PMC5207429; doi:10.1371/journal.pone.0168965)
Supplement: S5 Table — From top to the bottom represent the most stable to least stable gene. (DOCX) [file pone.0168965.s009.docx]

**S5 Table. Rankings and expression stability values of ten candidate reference genes in soybean leaves and roots under 100 μM CdCl_2_ treatment.** From top to the bottom represent the most stable to least stable gene.

| **RefFinder** | | **BestKeeper** | | **NormFinder** | | **Delta Ct** | | **geNorm(M)** | |
| --- | --- | --- | --- | --- | --- | --- | --- | --- | --- |
| *60S* | 1.190 | *UKN2* | 0.851 | *60S* | 0.274 | *60S* | 0.850 | *60S* | 0.424 |
| *UKN2* | 1.410 | *60S* | 1.115 | *UKN2* | 0.425 | *UKN2* | 0.900 | *UKN2* | 0.424 |
| *ACT2/7* | 3.000 | *ACT2/7* | 1.119 | *ACT2/7* | 0.490 | *ACT2/7* | 0.940 | *ACT2/7* | 0.509 |
| *ACT11* | 4.950 | *ABC* | 1.176 | *TUA4* | 0.580 | *TUA4* | 1.000 | *ACT11* | 0.638 |
| *TUA4* | 5.030 | *Fbox* | 1.265 | *ACT11* | 0.695 | *ACT11* | 1.030 | *TUA4* | 0.719 |
| *CYP2* | 6.240 | *ACT11* | 1.346 | *CYP2* | 0.729 | *CYP2* | 1.050 | *CYP2* | 0.787 |
| *ABC* | 6.730 | *CYP2* | 1.465 | *ELF1A* | 0.901 | *ELF1A* | 1.150 | *ELF1A* | 0.841 |
| *ELF1A* | 7.450 | *TUA4* | 1.628 | *ABC* | 0.939 | *ABC* | 1.170 | *ABC* | 0.920 |
| *Fbox* | 8.410 | *ELF1A* | 1.686 | *TUB4* | 1.190 | *TUB4* | 1.370 | *TUB4* | 1.003 |
| *TUB4* | 9.240 | *TUB4* | 1.952 | *Fbox* | 1.300 | *Fbox* | 1.430 | *Fbox* | 1.088 |
