# Supplementary material for: Evaluation of Reference Genes for Normalization of Gene Expression Using Quantitative RT-PCR under Aluminum, Cadmium, and Heat Stresses in Soybean
Source: PLoS One. 2017 Jan 3;12(1):e0168965. doi: 10.1371/journal.pone.0168965 (PMC5207429; doi:10.1371/journal.pone.0168965)
Supplement: S6 Table — From top to the bottom represent the most stable to least stable gene. (DOCX) [file pone.0168965.s010.docx]

**S6 Table.** **Rankings and expression stability values of ten candidate reference genes in soybean leaves under 42 °C heat stress.** From top to the bottom represent the most stable to least stable gene.

| **RefFinder** | | **BestKeeper** | | **NormFinder** | | **Delta Ct** | | **geNorm(M)** | |
| --- | --- | --- | --- | --- | --- | --- | --- | --- | --- |
| *UKN2* | 1.410 | *UKN2* | 0.332 | *UKN2* | 0.106 | *UKN2* | 0.700 | *60S* | 0.342 |
| *60S* | 1.860 | *ACT11* | 0.493 | *60S* | 0.282 | *60S* | 0.700 | *TUA4* | 0.342 |
| *TUA4* | 2.830 | *60S* | 0.498 | *ACT11* | 0.312 | *ACT11* | 0.760 | *ABC* | 0.383 |
| *ACT11* | 3.220 | *TUA4* | 0.672 | *TUA4* | 0.509 | *TUA4* | 0.800 | *UKN2* | 0.440 |
| *ACT2/7* | 5.000 | *ACT2/7* | 0.706 | *ACT2/7* | 0.595 | *ACT2/7* | 0.870 | *ACT2/7* | 0.522 |
| *ABC* | 5.450 | *ELF1A* | 0.773 | *ELF1A* | 0.694 | *ABC* | 0.880 | *ACT11* | 0.567 |
| *ELF1A* | 6.700 | *ABC* | 0.802 | *ABC* | 0.696 | *ELF1A* | 0.940 | *Fbox* | 0.625 |
| *Fbox* | 8.430 | *CYP2* | 0.829 | *TUB4* | 0.898 | *Fbox* | 1.070 | *ELF1A* | 0.720 |
| *TUB4* | 8.740 | *TUB4* | 0.943 | *Fbox* | 0.965 | *TUB4* | 1.070 | *TUB4* | 0.810 |
| *CYP2* | 9.460 | *Fbox* | 1.017 | *CYP2* | 1.200 | *CYP2* | 1.300 | *CYP2* | 0.909 |
