# Supplementary material for: Evaluation of Reference Genes for Normalization of Gene Expression Using Quantitative RT-PCR under Aluminum, Cadmium, and Heat Stresses in Soybean
Source: PLoS One. 2017 Jan 3;12(1):e0168965. doi: 10.1371/journal.pone.0168965 (PMC5207429; doi:10.1371/journal.pone.0168965)
Supplement: S7 Table — From top to the bottom represent the most stable to least stable gene. (DOCX) [file pone.0168965.s011.docx]

**S7 Table.** **Rankings and expression stability values of ten candidate reference genes in soybean roots under 42 °C heat stress.** From top to the bottom represent the most stable to least stable gene.

| **RefFinder** | | **BestKeeper** | | **NormFinder** | | **Delta Ct** | | **geNorm(M)** | |
| --- | --- | --- | --- | --- | --- | --- | --- | --- | --- |
| *TUA4* | 2.300 | *ACT11* | 0.429 | *TUA4* | 0.048 | *TUA4* | 0.390 | *CYP2* | 0.215 |
| *ACT2/7* | 3.310 | *TUB4* | 0.561 | *ACT2/7* | 0.134 | *ACT2/7* | 0.410 | *ELF1A* | 0.215 |
| *ELF1A* | 3.440 | *60S* | 0.606 | *UKN2* | 0.152 | *UKN2* | 0.420 | *ABC* | 0.236 |
| *UKN2* | 4.050 | *TUA4* | 0.673 | *ELF1A* | 0.348 | *ABC* | 0.480 | *Fbox* | 0.257 |
| *ABC* | 4.820 | *UKN2* | 0.704 | *ABC* | 0.365 | *ELF1A* | 0.480 | *ACT2/7* | 0.321 |
| *TUB4* | 4.900 | *ACT2/7* | 0.840 | *TUB4* | 0.403 | *TUB4* | 0.520 | *UKN2* | 0.353 |
| *CYP2* | 5.180 | *ELF1A* | 0.928 | *60S* | 0.420 | *Fbox* | 0.530 | *TUA4* | 0.365 |
| *ACT11* | 5.620 | *Fbox* | 0.954 | *Fbox* | 0.436 | *CYP2* | 0.530 | *TUB4* | 0.418 |
| *60S* | 6.420 | *ABC* | 0.990 | *CYP2* | 0.457 | *60S* | 0.540 | *60S* | 0.447 |
| *Fbox* | 6.510 | *CYP2* | 1.020 | *ACT11* | 0.677 | *ACT11* | 0.720 | *ACT11* | 0.502 |
