# Supplementary material for: Evaluation of Reference Genes for Normalization of Gene Expression Using Quantitative RT-PCR under Aluminum, Cadmium, and Heat Stresses in Soybean
Source: PLoS One. 2017 Jan 3;12(1):e0168965. doi: 10.1371/journal.pone.0168965 (PMC5207429; doi:10.1371/journal.pone.0168965)
Supplement: S8 Table — From top to the bottom represent the most stable to least stable gene. (DOCX) [file pone.0168965.s012.docx]

**S8 Table.** **Rankings and expression stability values of ten candidate reference genes in soybean leaves and roots under 42 °C heat stress.** From top to the bottom represent the most stable to least stable gene.

| **RefFinder** | | **BestKeeper** | | **NormFinder** | | **Delta Ct** | | **geNorm(M)** | |
| --- | --- | --- | --- | --- | --- | --- | --- | --- | --- |
| *UKN2* | 1.000 | *UKN2* | 0.794 | *UKN2* | 0.325 | *UKN2* | 0.820 | *UKN2* | 0.436 |
| *ACT2/7* | 2.280 | *Fbox* | 0.950 | *60S* | 0.353 | *60S* | 0.830 | *ACT2/7* | 0.436 |
| *60S* | 3.130 | *ACT2/7* | 0.995 | *ACT2/7* | 0.466 | *ACT2/7* | 0.880 | *ABC* | 0.510 |
| *ABC* | 4.160 | *ABC* | 1.030 | *ELF1A* | 0.557 | *ELF1A* | 0.930 | *60S* | 0.585 |
| *ELF1A* | 5.030 | *CYP2* | 1.038 | *ABC* | 0.655 | *ABC* | 0.960 | *ELF1A* | 0.703 |
| *ACT11* | 6.240 | *60S* | 1.055 | *ACT11* | 0.776 | *ACT11* | 1.020 | *ACT11* | 0.782 |
| *Fbox* | 6.690 | *ACT11* | 1.308 | *TUA4* | 0.827 | *TUA4* | 1.070 | *TUA4* | 0.824 |
| *TUA4* | 7.650 | *ELF1A* | 1.326 | *TUB4* | 0.928 | *TUB4* | 1.130 | *TUB4* | 0.869 |
| *CYP2* | 7.770 | *TUB4* | 1.394 | *CYP2* | 0.963 | *CYP2* | 1.190 | *CYP2* | 0.934 |
| *TUB4* | 8.240 | *TUA4* | 1.571 | *Fbox* | 1.244 | *Fbox* | 1.360 | *Fbox* | 1.020 |
